# The Potential of Harnessing IL-2-Mediated Immunosuppression to Prevent Pathogenic B Cell Responses

**DOI:** 10.3389/fimmu.2021.667342

**Published:** 2021-04-27

**Authors:** Amber Papillion, André Ballesteros-Tato

**Affiliations:** Division of Clinical Immunology and Rheumatology, Department of Medicine, University of Alabama at Birmingham, Birmingham, AL, United States

**Keywords:** IL-2 (interleukin-2), Tfh and immunity, autoimmune disease, Th17 & Tregs cells, auto-antibodies

## Abstract

Immunosuppressive drugs can partially control Antibody (Ab)-dependent pathology. However, these therapeutic regimens must be maintained for the patient’s lifetime, which is often associated with severe side effects. As research advances, our understanding of the cellular and molecular mechanisms underlying the development and maintenance of auto-reactive B cell responses has significantly advanced. As a result, novel immunotherapies aimed to restore immune tolerance and prevent disease progression in autoimmune patients are underway. In this regard, encouraging results from clinical and preclinical studies demonstrate that subcutaneous administration of low-doses of recombinant Interleukin-2 (r-IL2) has potent immunosuppressive effects in patients with autoimmune pathologies. Although the exact mechanism by which IL-2 induces immunosuppression remains unclear, the clinical benefits of the current IL-2-based immunotherapies are attributed to its effect on bolstering T regulatory (Treg) cells, which are known to suppress overactive immune responses. In addition to Tregs, however, rIL-2 also directly prevent the T follicular helper cells (Tfh), T helper 17 cells (Th17), and Double Negative (DN) T cell responses, which play critical roles in the development of autoimmune disorders and have the ability to help pathogenic B cells. Here we discuss the broader effects of rIL-2 immunotherapy and the potential of combining rIL-2 with other cytokine-based therapies to more efficiently target Tfh cells, Th17, and DN T cells and subsequently inhibit auto-antibody (ab) production in autoimmune patients.

## Introduction

Self-reactive auto-antibodies (auto-Abs) against nuclear and cytoplasmic antigens play critical roles in autoimmune disease development and severity (1, 2). Auto-Abs contribute to disease pathogenesis by direct and indirect mechanisms. On the one hand, immune complexes (IC) formed by Auto-Abs and self-antigens activate antigens presenting cells and innate cells through the activation of Fc receptors (FCRs), thereby initiating a feedback loop of immune activation that ultimately leads to unwarranted inflammation and tolerance breakdown (3–5). Auto-Abs also engage the complement system, which mediates tissue damage and further contributes to triggering systemic inflammation. Deposition of IC in the blood vessels, kidney, joints, and lungs amplifies the local inflammatory response and enhance tissue damage. In agreement with their pathogenic roles, the serum levels of auto-Abs strongly correlate with disease activity and severity in multiple forms of autoimmune disease, including systemic lupus erythematosus (SLE), type 1 diabetes (T1D), or rheumatoid arthritis (RA), among others. Furthermore, Auto-Abs can be detected years before the onset of clinical manifestations (6), thus suggesting that loss of B cell tolerance and production of Auto-Abs is a critical step that precedes the development of the autoimmune disease. While Ab-dependent pathology can be partially controlled by immunosuppression, there is currently no cure for systemic autoimmune disorders.

Auto-Abs are produced by autoreactive-plasma cells (PCs), a subset of terminally differentiated B cells that secrete large amounts of Abs (7). PCs can be originated in the germinal centers (GCs), the site of B cell maturation, where B cells undergo rapid rounds of proliferation, somatic hypermutation, and affinity maturation leading to the generation of high-affinity antibodies (8–10). Autoreactive PCs can also be generated outside the GCs *via* the extrafollicular pathway (11–13). Recent studies demonstrate a critical role for the extrafollicular PCs in the development of pathogenic Ab responses (11, 13, 14).

**Figure 1:**
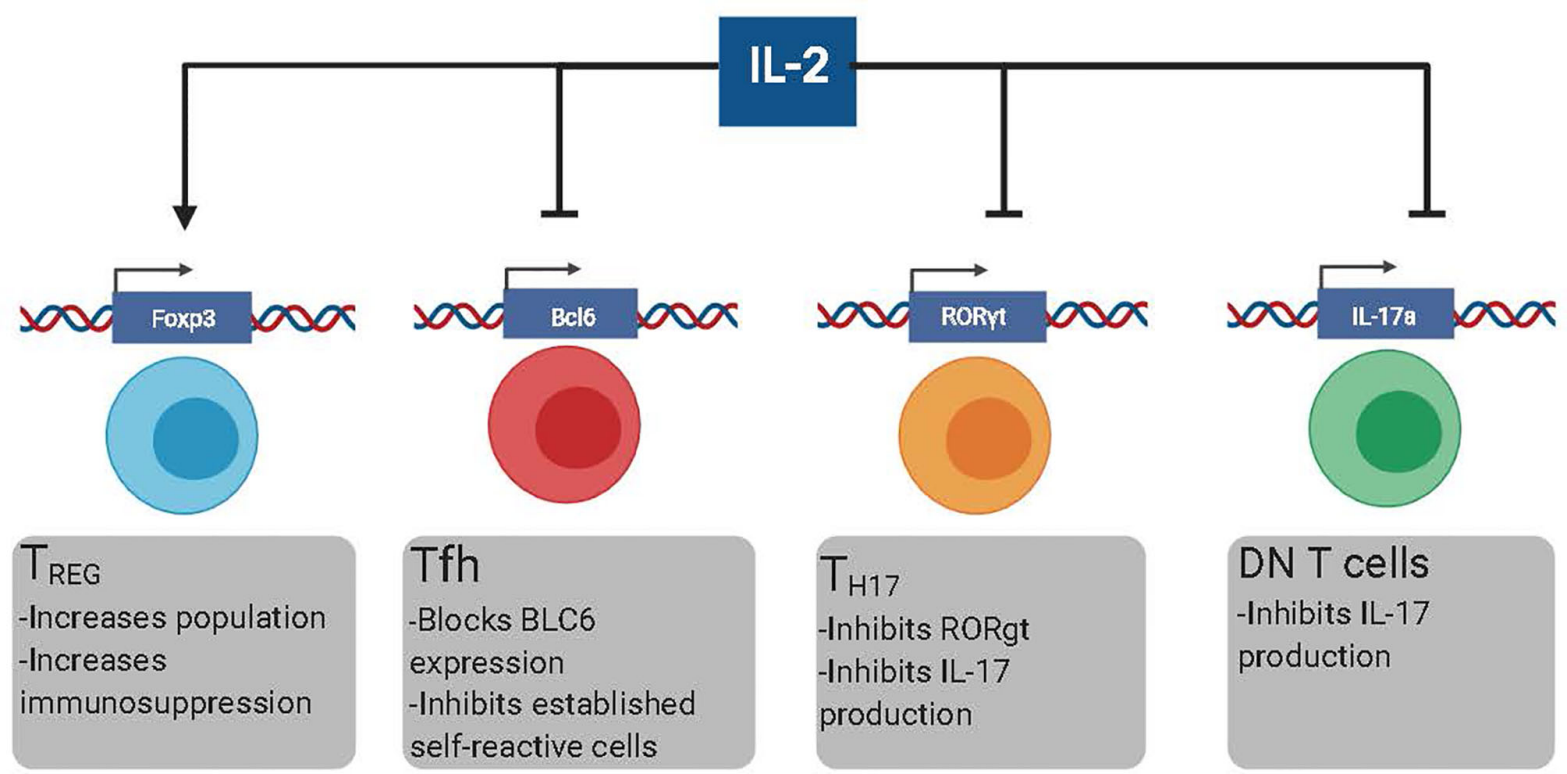
The different effects of low dose rIL-2 therapy in autoimmunity. Low dose rIL-2 stabilizes FoxP3 program in Treg cells which increases both the size of the population and enhances immunosuppression. Low dose rIL-2 therapy can both inhibit the generation of new self-reactive Tfh cells and decrease already present self-reactive Tfh cells by blocking Bcl6. IL-6 blockade will make Tfh cells more suspectable to IL-2 signaling. Low dose rIL-2 can inhibit Th17 cells by diminishing expression of RORgt and inhibiting *IL17a* expression. Low dose rIL2 can also inhibit IL-17 production by DN T cells by directly inhibiting *IL17a*.

In the last decade, B cell depleting therapies were designed based on the rationale that depletion of self-reactive B cells would reduce the production of auto-Ab and subsequent Auto-Ab-mediated immunopathology (15, 16). However, the clinical efficacy of these therapies is lower than initially anticipated (17–19). The inability of B cell depleting agents to eliminate self-reactive PCs efficiently has been suggested as a plausible explanation for the relatively low effectiveness of these approaches (17). The life-threatening side effects of sustained immunosuppression and the failure of new therapies, such as B cell depletion, vindicate looking for new therapeutic alternatives to treat Ab-mediated pathologies. In this manuscript, we review the potential of low-dose IL-2-based immunotherapies to target T cell populations with B cell helper activity, mainly T follicular helper cells (Tfh), T helper 17 (Th17) cells, and Double-negative (DN) CD3+CD4-CD8- T cells (**Figure 1**). While IL-2 also induces immunosuppression by Treg-dependent mechanisms, more extensive reviews on this topic are available elsewhere. Hence, the role of IL-2 in promoting Treg-mediated immunosuppression will be only briefly discussed in this review.

## Pathogenic B Cell Helper T Cell Subsets

### Tfh Cells

**Figure 2:**
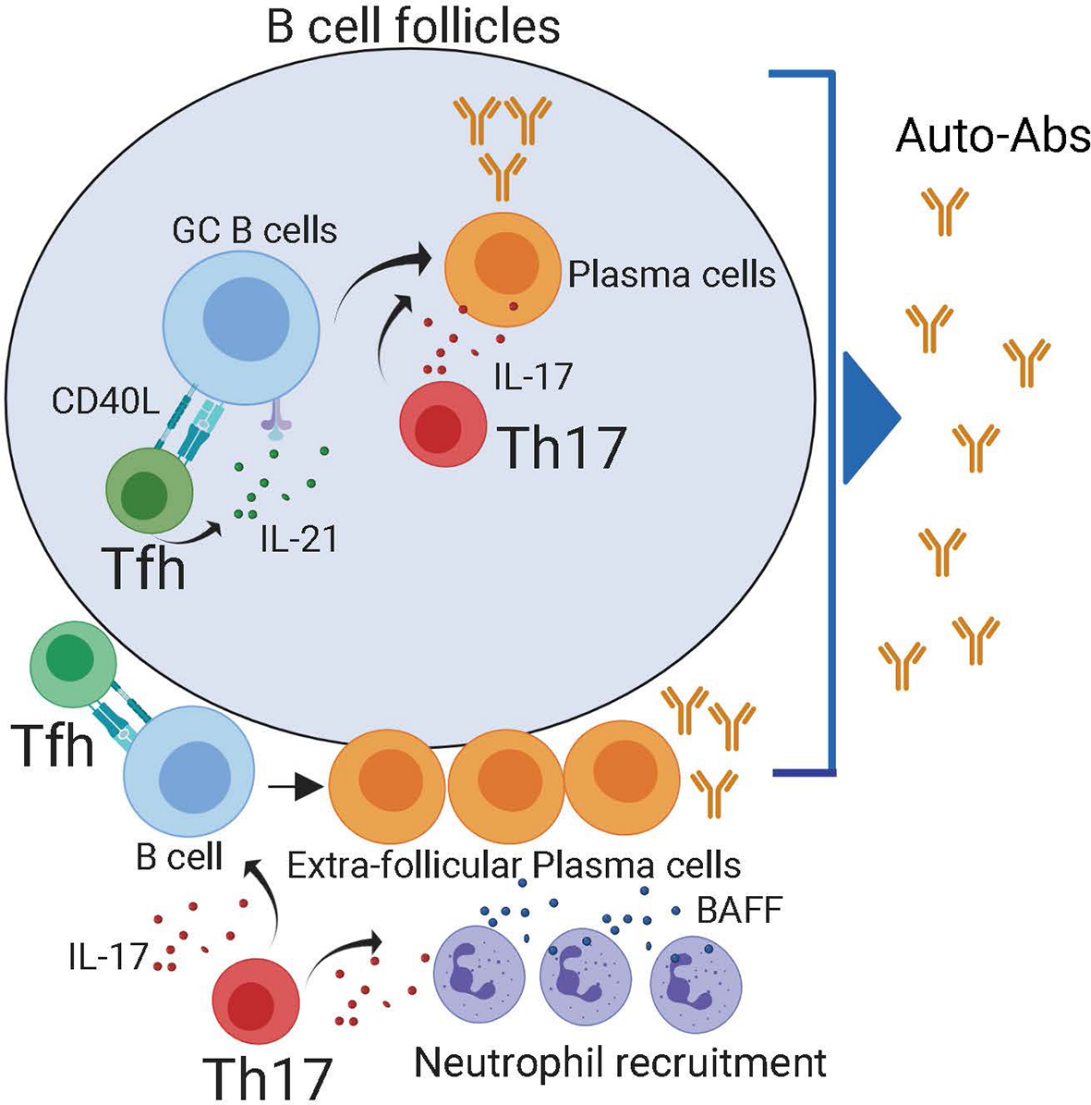
Both follicular and extra-follicular pathways contribute to auto-antibody production. Within the B cell follicles, Tfh cells maintain auto-reactive germinal center reactions which generate self-reactive plasma cells. Tfh cells can also provide help in to extra-follicular PCs in the B cell border. Reports have demonstrated that IL-21 produced by Th17 cells can also contribute to the production of self-reactive plasma cells in the follicle. Besides, in the extra-follicular space, IL-17 produced by Th17 recruits BAFF producing neutrophils. The combination of IL-17, BAFF, and Tfh cells promotes the generation of self-reactive plasma cells.

T follicular helper (Tfh) cells are a subset of CD4^+^ T cells that provide co-stimulatory signals and cytokines that are required for the development and maintenance of GCs (20–22) and extrafollicular PC differentiation (23, 24) **(** **Figure 2** **)**. In the absence of pathogen-specific Tfh cells, GCs do not develop, and pathogen-specific PC responses are impaired. Phenotypically, Tfh cells are characterized by the expression of CXCR5, PD1, ICOS, and Bcl6, among other markers (25). CXCR5 is a chemokine receptor that allows Tfh cells to localize in the proximity of the B/T cell border in response to CXCL13. The inhibitory receptor PD-1 and the co-stimulatory receptor ICOS ultimately direct Tfh cells into the B cell follicles (26–28), where they provide CD40L (22, 29) and IL-21 (30–32) to the responding B cells. Bcl6 is a transcription factor that promotes the expression of genes required for Tfh cell development and function while preventing the up-regulation of transcription factors implicated in T effector (Teff) cell differentiation (33–35). Bcl6 is critical for the differentiation of Tfh cells (33–35). Thus, it is considered the master regulator of Tfh differentiation. While Bcl6 promotes Tfh formation, the transcription factor Blimp-1 represses it (33–35). Importantly, Blimp-1 and Bcl6 are mutually antagonistic transcription factors that directly repress one another in CD4^+^ T cells. Thus, the balance between the relative expression of Bcl6 and Blimp-1, rather than the expression of Bcl6 alone, fine-tunes the commitment into the Tfh cell pathway (20).

Under homeostatic conditions, Tfh cells help germinal center B cells to facilitate somatic hypermutation and class switch to generate long-term Ab protection to pathogens (25). However, when there is a break in intolerance, self-reactive Tfh cells are not depleted from the repertoire and provide co-stimulatory signals and cytokines to self-reactive B cells, leading to pathogenic auto-Ab responses (36, 37). In agreement, the presence of Tfh cells correlates with elevated levels of Auto-Ab and disease activity in preclinical animal models and autoimmune patients (37–41). When Tfh cells are depleted or decreased, autoimmune disease pathogenesis and auto-Ab responses are reduced (40, 42, 43). Based on these findings, Tfh cells are considered a potential target for autoimmune disorders (44). However, to date, there are no therapeutic agents approved to selectively deplete Tfh cells *in vivo.*

### Th17 Cells

Th17 cells are a specialized subset of CD4^+^ T cells that play an essential role in aiding host defense by recruiting neutrophils and macrophages. Th17 cells differentiate in response to TGFβ and IL-6, and their development is driven by the transcription factor RORγt (45). Excessive Th17 cell responses are implicated in the pathogenesis of multiple forms of autoimmune diseases. As such, the expansion of self-reactive Th17 cells correlates with disease activity in common autoimmune diseases, including RA, psoriasis, asthma, and lupus (46–48). Due to its characteristic pro-inflammatory properties, it is generally believed that Th17 cells contribute to autoimmune disease pathogenesis by inducing tissue inflammation. However, recent studies demonstrate that Th17 cells can also help the development of auto-reactive GC B cells (48–51) and extrafollicular PCs (52). In agreement with this idea, IL-17 deficiency prevents auto-Ab production and disease progression in lupus-prone mice (53–55). In addition, the self-reactive GC responses were reduced by IL-17 deficiency in autoimmune Roquin^san/san^ mice, thereby suggesting a cause-effect relationship between IL-17 and pathogenic GC B cell responses. Collectively, these studies suggest an important role for IL-17 producing cells in promoting pathogenic-B cell responses in the context of autoimmune disorders.

The capacity of Th17 cells to help B cell responses is not entirely surprising since, like Tfh cells, Th17 characteristically produce large amounts of IL-21 (56), a cytokine that promotes GC and PC differentiation. Furthermore, IL-17 synergizes with the B cell-activating factor belonging to the TNF family, BAFF, to protect responding cells from BCR-induced apoptosis (57), demonstrating an intrinsic effect of IL-17 in promoting B cell survival. Besides, IL-17 promotes the recruitment of neutrophils (58, 59), which secrete both BAFF and APRIL and can facilitate the survival and devolvement of extrafollicular PCs (60, 61). Collectively, these studies suggest that IL-17-producing cells can, directly and indirectly, help auto-reactive B cells responses, thereby contributing to Ab-mediated pathology in autoimmune patients **(** **Figure 2** **)**. However, whether IL-17-producing cells promote rather than merely correlate with self-reactive B cell responses in autoimmune patients has not yet been formally demonstrated.

### Hybrid IL-17^+^Tfh Cells?

An additional important question remaining is how the putative *“IL-17^+^ helper”* cells gain access to the B cell follicles to provide B cell help. One exciting possibility is that pathogenic *“IL-17^+^ helper”* cells are indeed Tfh cells that secrete IL-17. In agreement with this possibility, studies suggest the presence of hybrid IL-17-producing cells with “Tfh-like” characteristics in autoimmune prone BXD2 mice (48) and human tonsils (45). Furthermore, the culture of human CD4^+^ T cells with a combination of TGFβ and IL-23, which is frequently used for the *in vitro* differentiation of Th17 cells (45), triggers the acquisition of a Tfh-like transcriptional signature characterized by the up-regulation of Bcl6, c-Maf, and CXCR5, and the down-regulation of Blimp-1, thereby resulting in the acquisition of a hybrid Bcl6^+^RORγt^+^ Tfh/Th17 signature (62). Whether hybrid Tfh/Th17 cells are Tfh cells that secondary acquire the capacity of secrete IL-17 or represent a separate lineage of Tfh cells is still unclear. Further investigations are needed in order to clarify the potential relationship between these two lineages.

Interestingly, the ‘pro-Tfh” effect of TGF-β is restricted to humans, as TGF-β does not significantly affect Tfh cell differentiation in mice (62, 63). Nevertheless, the commonality between the Tfh and Th17 differentiation requirements extends beyond TGF-β. For example, ICOS, which is required for the survival and the migration of Tfh cells into the B cell follicles (26, 28, 64), is also critical for the differentiation and maintenance of Th17 cells (65, 66). Besides, similar to Tfh cells, the IL-6/STAT3 pathway is also a key positive regulator of Th17 differentiation (67). Thus, critical signaling pathways implicated in Tfh cell differentiation also critically regulate the Th17 program. Therefore, it is reasonable to speculate that the same inflammatory conditions that promote Tfh differentiation in autoimmune patients also favor the development of Th17 cells and/or the generation of hybrid IL-17-producing Tfh cells.

The concept of a highly pathogenic hybrid Tfh/IL-17 population with superior helper activity is, however, at odds with early studies suggesting that Bcl6 functions as a direct transcriptional repressor that prevents the acquisition of Teff programs, including the Th17 program (33–35). Indeed, Bcl6^hi^CXCR5^hi^PD-1^hi^ Tfh cells present in the B cell follicles (we will refer to these cells as GC-Tfh cells) do not normally produce IL-17, which is consistent with studies showing that high-expression of Bcl6 in GC-Tfh cells directly represses RORγt and Th17 differentiation (33, 34). Nevertheless, the role of Bcl6 in controlling alternative differentiation programs in Tfh cells is puzzling. As such, while studies suggest that Bcl6 binds to the *Rorc* promoter and inhibits its expression (33), other studies show no evidence of Bcl6 binding (34, 68). Furthermore, while Bcl6-expressing cells do not normally express Teff cytokines, some studies indicate that Tfh cells can produce effector cytokines in the context of high inflammatory conditions, such as viral infections (69–72) or autoimmune diseases (48). In addition, extrafollicular-Tfh cells (which express medium levels of Bcl6) have a more heterogeneous transcriptional signature than Bcl6^hi^ GC-Tfh cells (which express high levels of Bcl6) (73–75). These results suggest that the ability of Bcl6 to inhibit the initiation of secondary Teff differentiation programs in developing Tfh cells is dose-dependent and can be partially overcome in highly reactive environments, such as in autoimmune diseases, thereby leading to the acquisition of hybrid Tfh/Teff phenotypes, such as IL-17^+^ Tfh cells, with enhanced pathogenic functions.

### Double-Negative T Cells

Double-negative (DN) CD3^+^CD4^-^CD8^-^ T cells are a rare population of TCR-αβ^+^ T cells that lack CD4 and CD8 expression and express high levels of B220 (76, 77). While DN T cells are relatively scarce in healthy individuals, they abnormally expand in lupus patients and children with autoimmune diseases, such as mixed connective tissue disease or juvenile idiopathic arthritis (78). Aberrant accumulation of DN T cells is also a clinical hallmark of the Autoimmune Lymphoproliferative Syndrome (ALPS, also known as Canale-Smith syndrome), a genetic disorder caused by defective FAS-mediated apoptosis that is characterized by the development of autoimmune disease, splenomegaly, lymphadenopathy, and an increased risk of secondary lymphomas during childhood (79, 80). In aged MRL/*lpr* mice, DN T cells represent nearly 70% of the total cells in the enlarged lymph nodes, accounting for the characteristic lymphadenopathy observed in these mice.

The exact origin of DN T cells remains controversial (76, 77). Early studies suggest they derive from activated CD4 T cells that fail to undergo apoptosis (81). However, a more detailed examination of the DN T cell origin *in vivo* indicates that DN T cells derive from CD8^+^ T cells that down-regulate their co-receptor after continuous stimulation by self-antigens derived from apoptotic cells (82, 83). Thus, it is generally believed that DN T cells derive from CD8^+^ T cells.

Expansion of DN T cells correlates with disease activity in lupus-prone mice (83, 84) and systemic SLE patients (85), leading to the idea that these cells play an important pathogenic role in autoimmune disease development. Despite the evidence supporting a pathogenic role for DN T cells, their exact function remains largely elusive (76, 77). Interestingly, DN T cells express high levels of CXCR5, localize in the B cells follicles (86), and stimulate Ab production *in vitro* (85, 87). Moreover, similar to Tfh cells, the presence of DN T cells correlates with disease activity and autoantibody production in SLE patients and MRL.lpr mice (76, 77, 83–85, 88). Moreover, new studies show that DN T cells produce large amounts of IL-17 (54, 83, 88). Given the putative role of IL-17 in helping B cell responses (48–52, 56, 89), it is tempting to speculate that CXCR5^+^DN T cells contribute to autoimmune pathology by promoting auto-reactive B cell responses in an IL-17-dependent manner. Corresponding with this idea, a recent study demonstrated that DN T cells are sufficient to promote autoantibody production and renal immune complex deposition after adoptive transfer into B6 *Rag1* ^−^ *^/^* ^−^ mice that also received B cells from 12-month-old B6.*lpr* mice (83). These findings provide evidence that DN T cells can contribute to pathogenic Ab responses *in vivo.* Further investigations will be required to compare the capacity of DN T cells, Tfh cells, and bona fide Th17 to help self-reactive B cell responses and determine how each of these subsets relatively contribute to sustaining pathogenic-Ab responses.

## IL-2 and Immunosuppression

### IL-2 Signaling

IL-2 is a member of the common γ-chain family of cytokines that was initially characterized and as a growth factor for T and NK T cells (90–92). IL-2 signaling is transmitted through the IL-2 receptor (IL-2R), which can exist in two conformations** ** (93). The high-affinity receptor is a heterotrimeric receptor that consists of the α chain (CD25), the β chain (CD122), and the common γ chain (CD132)** ** (94, 95). The high-affinity receptor is constitutively expressed by FoxP3-expressing CD4^+^ regulatory T-cells (Tregs), which require IL-2 signaling for their differentiation and function** ** (96–99). In contrast, NK T cells, naïve and memory T cells express the intermediate-affinity IL-2R, a heterodimer composed of the β and γ chain. Following TCR activation, however, they transiently up-regulate CD25 and temporarily express the high-affinity IL-2R. The differential expression of CD25 by regulatory T cells and conventional T cells has important therapeutic consequences. When administered at high doses, IL-2 can help conventional T cells and NK T cells, hence favoring effector responses. In contrast, because Tregs express high levels of CD25 and better compete for IL-2 than other cells, low IL-2 regimes preferentially target IL-2 to Tregs, thus promoting immunosuppression (100).

The binding of IL-2 to the IL-2R triggers the phosphorylation of the Janus-Activated Kinase 1 (JAK1) and 3 (JAK3), leading to the activation of the transcription factor STAT5 (101). In addition, phosphorylation of the adaptor Shc in response to IL-2 activates the Ras-Raf MAP Kinase and PI-3K pathways. The combined effects of STAT5, Ras-Raf MAP Kinase, and PI-3K signaling pathways results in the regulation of the transcription of a broad range of IL-2-target genes, including the forkhead box P3 (FOXP3) (102–104), eomesodermin (Eomes) (105), the B Lymphocyte Induced Maturation Protein 1 (BLIMP1) (105), the T-box transcription factor TBX21 (T-bet) (106, 107), Retinoic acid-related Orphan Receptor (ROR)γ (108–110) and B cell lymphoma (Bcl6) (107, 111–114). Due to its pleiotropic transcriptional effects, IL-2 has been implicated in regulating multiple, and often contradictory, critical immunoregulatory pathways. For example, IL-2–STAT5 signaling positively regulates IL-4R and GATA-3 expression and subsequent Th2 differentiation (106). On the other hand, IL-2 induces IL-12Rβ2, Blimp-1, and IFN-γ up-regulation, which are required for Th1 cell polarization (106).

Importantly, while IL-2 signaling can help effector responses, the development of a lethal multiorgan autoimmune syndrome in the IL-2 and IL-2R deficient mice revealed that the critical non-redundant function of IL-2 is to promote immunosuppression (115–118). Rather than immunodeficiency, diminished IL-2 production is associated with autoimmune disease development in mice and humans (119–123), highlighting the critical role of this cytokine in maintaining immunological tolerance. Given that Treg cells fail to normally develop in the absence of IL-2 signaling (98, 124–126) and that they are essential for maintaining immune tolerance (127–130), it is generally accepted that the principal mechanism by which IL-2 contributes to preserving immune tolerance is by supporting the development and function of Tregs. Supporting this view, early transfer of Tregs into neonatal CD122 deficient mice prevents autoimmune pathology (96).

### Low-Dose IL-2 Therapy

Work done over the last fifteen years demonstrate the potential of leveraging the immunosuppressive properties of IL-2 to treat autoimmune disorders. Early studies show that exogenous IL-2 supplementation prevents disease progression and contributes to inducing immunosuppression in mice with established autoimmune diseases, including Type I diabetes, EA, experimental myasthenia, and lupus (131–138). More recently, a novel immunotherapy based on subcutaneous administration of low-dose recombinant human IL-2 (r-IL2, (Aldesleukin/Proleukin) has shown potent immunosuppressive effects in patients with autoimmune pathologies (139), including Type I diabetes (140), hepatitis C-associated vasculitis (141), SLE (142–145), and chronic graft-versus-host disease (146–148). The recent TRANSREG clinical trial further demonstrated that the same dose of rIL-2 selectively expands Tregs and clinical benefits across eleven selected autoimmune diseases (149). Collectively these studies demonstrate that low-dose rIL-2 regimes have therapeutic effects across a broad range of heterogonous autoimmune disorders.

Current low-dose rIL-2 treatment schemes consist of 3-4 cycles of 7-10 million IU of rIL-2 per cycle administered over 1-2 weeks separated by resting periods of 9-16 days. Importantly, low-dose rIL-2 can be safely administered to humans. Thus numerous clinical trials to further explore the potential benefits of low-dose IL-2 in SLE are now underway.

Based on the critical functional relationship between IL-2 and Treg-mediated immunosuppression, the current paradigm suggests that low-dose rIL-2 regimes contribute to restoring immune homeostasis in autoimmune patients by a Treg-dependent mechanism (139, 150). In agreement with this view, low-dose rIL-2 supplementation induces Treg cell expansion *in vivo* (139). Hence considerable effort has been invested in developing new therapeutic approaches to selectively target IL-2 to Tregs.

Intriguingly, though the frequency of Tregs increases after low-dose rIL-2 administration, the changes in Treg cell numbers are transient and drop to placebo control levels quickly after the last rIL-2 cycle (142, 143). Nevertheless, despite a nearly normal frequency of Tregs, the improved clinical outcomes persist for weeks after the last cycle of rIL-2 (142, 143). Thus, while it is clear that Treg-mediated immunosuppression is critical for achieving the clinical benefits observed after rIL-2 treatment, additional underlying mechanisms might synergize with Treg-mediated effects to provide long-lasting immunosuppression after low-dose rIL-2 immunotherapy. In this regard, recent studies demonstrate that prolonged IL-2 signaling prevents the expression of (RORγt) (110) and Bcl6 (107, 111–114), thereby repressing Th17 and Tfh cell development, respectively. Correspondingly, low-dose rIL-2 treatment significantly reduced the frequency of Tfh and Th17 in humans and preclinical animal models (71, 114, 138, 143, 151, 152). Similarly, CD3^+^CD4^-^CD8^-^ DN T cells are depleted after rIL-2 administration (138). Based on the inhibitory role of IL-2 in Tfh, Th17, and DN T cells and their putative roles in promoting self-reactive B cell responses, targeting IL-2 to these T cell populations could represent a good therapeutic strategy to prevented Ab-mediated pathology in autoimmune patients without inducing profound immunosuppression.

## Targeting B Cell Helpers with IL-2

### IL-2 and Tfh Cells

Studies by us and others demonstrate that IL-2 signaling inhibits Tfh cell differentiation (107, 111, 113, 114, 152). Mechanistically, IL-2 indirectly inhibits Tfh cells by inducing BLIMP, which in turn represses Bcl6 expression and Tfh cell differentiation (111, 113). Besides, STAT5 in response to IL-2 binds to the Bcl6 promoter and directly prevents Bcl6 transcription (107, 112), thereby inhibiting the initiation of the Tfh cell program. In support of these findings, the lack of IL-2/STAT5 signaling during T cell differentiation skews the CD4^+^ T cell response towards the Tfh cell differentiation pathway (111, 113, 114). Data from the Weinmann’s laboratory also suggest that, in addition to directly repressing Bcl6 expression, IL-2 signaling favors the formation of T-bet/Bcl6 complexes that block Bcl6 activity (107).

Corresponding with the inhibitory role of IL-2 in Tfh cell development, Tfh cell differentiation can be fine-tuned *in vivo* by altering the environmental levels of IL-2. As such, limiting IL-2 signaling *in vivo* results in enhanced Tfh cell responses (111, 113, 114, 151–153). Contrariwise, treatment with rIL-2 prevents Tfh cell differentiation and ensuing GC responses in mice infected with influenza virus (71, 114, 151). Importantly, in these studies, the ability of IL-2 to suppress Tfh cell responses is independent of the presence of Tregs (114, 152). Hence, these studies demonstrate that lL-2 intrinsically inhibits Tfh cell development by repressing Bcl6 expression and activity in a Treg-independent manner.

Notably, there are significant differences between human and mouse Tfh cell developmental requirements (154, 155). However, recent studies demonstrate that IL-2 is also a potent inhibitor of human Tfh cell responses. Corresponding with this, while IL-2 blockade increases human Tfh cell differentiation *in vitro* (156), treatment with low-dose rIL-2 reduces the frequency of Tfh cells in SLE patients (143). These results provide evidence to support the potential of IL-2-based therapies to deplete Tfh cells *in vivo*. Furthermore, these studies offer a new interpretation for how impaired IL-2 production by T cells (120–123) and single nucleotide polymorphisms in the IL-2 and IL-2 receptor genes (122, 157, 158), which are associated with various autoimmune diseases, affects autoimmune disease development. In this regard, one would predict that a low IL-2 environment favors self-reactive Tfh cell differentiation and subsequent Auto-Ab production in autoimmune patients.

### Synergistic Low-Dose rIL-2 Therapies

**Figure 3:**
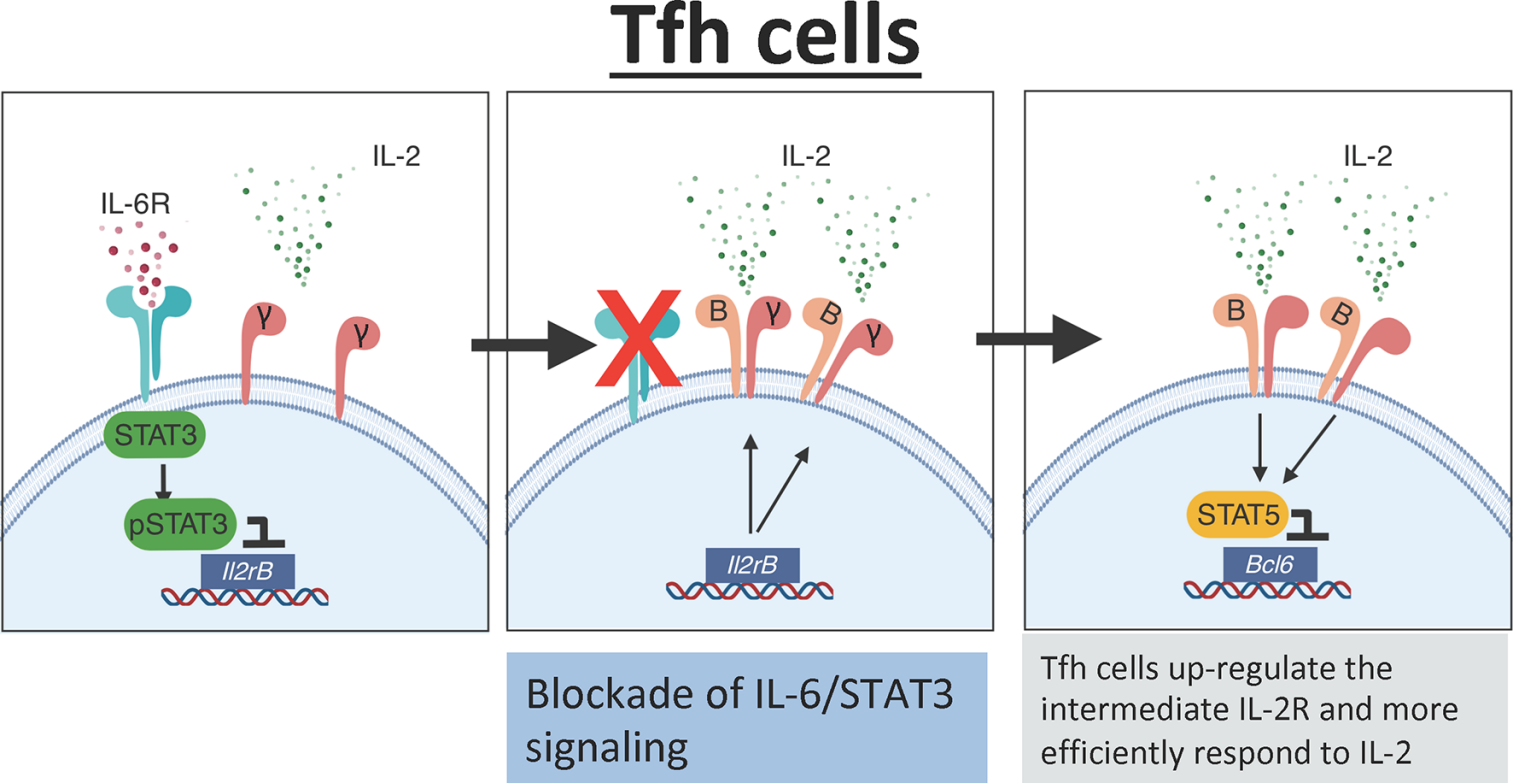
IL-6 mediates the IL-2 responsiveness of Tfh cells. In the presence of IL-6 signaling, Tfh cells activate STAT3 which directly binds to the Il2rb promoter preventing expression of CD122. Low expression of CD122 limits IL-2 signaling in Tfh cells. In the absence of IL-6 signaling, there is increased expression of CD122 on the surface of Tfh cells leading to increased responsiveness to IL-2. The increased IL-2 signaling activates STAT5 which binds to the promoter region of Bcl6 and suppresses the Tfh cell program.

Recent studies suggest that in addition to secreting a low amount of IL-2, T cells from SLE patients poorly respond to exogenous IL-2 (159). Thus, the lack of IL-2 responsiveness could be a potential limitation when designing low-IL-2-based therapies to efficiently deplete Tfh cells *in vivo*. Importantly, data from our laboratory demonstrate that the IL-6/STAT3 pathway is an important regulator of the IL-2 responsiveness of Tfh cells. Briefly, using a combination of *in vivo* and genetic studies, we found that STAT3 in response to IL-6 binds to the *Il2rβ* locus and prevents CD122 up-regulation in Tfh cells, thereby limiting the capacity of these cells to respond to IL-2 (151). Hence, blockade of IL-6 signaling renders Tfh cells hyperresponsive to IL-2, thus lowering the threshold of IL-2 required to deplete Tfh cells (**Figure 3**). As a consequence, the frequency of Tfh cells was dramatically reduced in influenza-infected mice treated with an anti-IL-6 blockade in combination with rIL-2 compared to mice treated with rIL-2 alone, even when rIL-2 was administered at ultra-low doses.

These findings have important therapeutic and conceptual implications. In this regard, one would predict that *Tocilizumab*, a humanized anti-IL-6 receptor monoclonal antibody, administered together with rIL-2 will synergize to target the Tfh cell population more efficiently than rIL-2 administered alone. In this scenario, an IL-6 blockade would increase the expression of CD122 on the surface of Tfh cells, making them more susceptible to rIL-2 signaling and subsequent depletion. This combination therapy will likely achieve the same biological effect using a lower dose of rIL-2. In addition, IL-2 consumption by CD25^+^Tregs limits the amount of available IL-2 (152, 160). Thus, it is likely that, in the presence of high numbers of CD25+Tregs, the environmental levels of IL-2 are scarce, and only cells with a relatively low response threshold will be able to respond to IL-2. Hence, another significant advantage of this approach is that, by increasing IL-2 responsiveness, this synergistic therapy will allow Tfh cells to react to rIL-2 even in the presence of IL-2-consuming Tregs, thereby simultaneously targeting Tregs and Tfh cells.

Importantly, similar to IL-6 (161, 162), the serum levels of the STAT3-activating cytokines IL-23 and IL-27 are increased in autoimmune patients (163–167). Thus, IL-6 signaling blockade alone might not be sufficient to enhance IL-2 responsiveness of Tfh cells due to the capacity of additional STAT3-activating cytokines to compensate for the lack of IL-6. Furthermore, IL-6, IL-23, and IL-27 all induce STAT3 activation *via* JAK2. In contrast, JAK2 is not required for IL-2 signaling. Notably, a recent study shows that the specific JAK2 inhibitor CEP-33779 can be safely administered to MRL.lpr mice, which show significant improvement in disease pathogenesis and reduced pSTAT3 levels after treatment (168). Given that JAK2 is required for STAT3 but not STAT5 activation, it is tempting to speculate that “*non-cytokine specific*” STAT3 inhibition after treatment with a JAK2 inhibitor will lower the threshold of IL-2 required for suppressing Tfh cells regardless of the presence of redundant STAT3-activating cytokines. In any case, altogether, these studies suggest a model in which STAT3 activation in response to STAT3-activating cytokines counterbalances IL-2-mediated suppression of Tfh cells by limiting IL-2 responsiveness of Tfh cells. A better knowledge of how the crosstalk between different cytokine pathways regulates Tfh cell development will allow us to design more efficient therapeutic strategies to prevent self-reactive Tfh cell responses in autoimmune patients, thereby precluding ensuing pathogenic B cells responses and Ab-mediated pathology.

### IL-2 and Th17 Cells

The clinical benefits of targeting Th17 cells to prevent autoimmune manifestations have been explored in preclinical and clinical settings, and additional clinical trials are being conducted. The results, however, are conflicting (169–171). Independent randomized clinical trials demonstrate the clinical efficacy of targeting IL-17 to treat moderate to severe psoriasis. As a result, two monoclonal anti-IL-17A antibodies (secukinumab and ixekizumab) and one antibody targeting the IL-17 receptor (brodalumab) are now FDA approved for the treatment of this disease. However, the studies assessing the clinical benefits of anti-IL-17 biologics for the treatment of systemic rheumatologic disorders, such as RA or SLE, have yielded mixed results. While some preclinical studies and clinical trials show promising results after IL-17 blockade (169), the therapeutic effect of anti-IL-17 anti-IL-17A Abs is lower than anticipated.

The relatively low efficacy of these treatments is, to some extent, surprising, given the abundance of publications showing reduced pathology and severity after IL-17 blockade in preclinical animal models (172). One potential explanation for this discrepancy is patient sample heterogeneity. In this regard, while most of the studies show that elevated levels of IL-17 and high frequency of Th17 cells correlate with disease activity in SLE patients, some found no significant differences between patients and healthy controls  (172). Since anti-IL-17 biological-based treatments will likely only be effective in patients with a “high IL-17” profile, the lack of proper patient stratification based on their IL-17 profile could explain the lack of consistency in the results. An alternative, but mutually complementary, explanation is the inability of the current anti-IL-17 biologics to sufficiently block the aberrantly increased IL-17 pathway in these patients. Besides, IL-17/IL-17A blockade alone might not be sufficient to effectively disrupt the inflammatory cycle leading to disease pathogenesis once this has already been initiated.

As aforementioned, IL-2 signaling inhibits Th17 differentiation (106, 108, 110). As a consequence of this inhibitory effect, Th17 cells fail to differentiate in relatively high IL-2 environments. Contrariwise, IL-2 quenching facilitates Th17 cell development (109). Mechanistically, IL-2 antagonizes IL-17 differentiation *via* STAT5, which outcompetes STAT3 binding at the IL-17 locus, hence preventing binding of STAT3 and its enhancer elements in response to IL-6 (108). IL-2 signaling also represses IL-6R expression and recruits the histone deacetylator adaptor protein NCoR2 to the *Il17* locus, thereby contributing to further inhibiting IL-17 production (108). In agreement with the suppressor role of IL-2 in Th17 cell differentiation, the regulatory mechanisms that control IL-2 production also indirectly control IL-17 production. For example, the cAMP-responsive element modulator alpha (CREMα) negatively regulates IL-2 transcription by binding to the *Il2* locus (121, 173–175). IL-2 shortage after CREMα overexpression in T cells contributes to enhancing IL-17 differentiation, a phenomenon that can be reserved after IL-2 supplementation (175). Similarly, by suppressing IL-2 production, the phosphatase and tensin homologue (PTEN) indirectly favors Th17 differentiation (176). At a cellular level, IL-2 consumption by Treg cells favors Th17 development by creating a low IL-2 environment permissive for Th17 differentiation (160). Collectively, these studies demonstrate that Th17 cells preferably differentiate in “low-IL-2” environments.

Importantly, works from multiple laboratories demonstrate a causative relationship between IL-2 deficiency, subsequent excessive IL-17 responses, and autoimmune pathology development. For example, elegant work has shown that a lack of STAT3 activation prevents the accumulation of Th17 cells in IL-2-deficient mice, resulting in prolonged lifespan and reduced autoimmunity associated with IL-2 deficiency (108). Additional studies have shown that MRL/Fas(lpr/lpr) mice treated with rIL-2 have reduced frequency of IL-17 producing cells, which correlated with diminished disease manifestations (138). Moreover, in SLE patients, low-dose rIL-2 treatment resulted in reduced frequencies of Th17 cells, which correlated with the induction of remission in a recent open-labeled trial (143). Collectively, these results provide strong evidence for the therapeutic potential of rIL-2 to prevent unwanted Th17 responses *in vivo*. Importantly, current anti-IL-17 biologics target the product of Th17 cells (i.e., IL-17). In contrast, low-dose rIL-2 precludes the development of these cells, which has the potential to more effectively prevent IL-17-dependent immunopathology by preventing the continuous replenishment of Th17 cells from their precursors, thereby inducing long-lasting effects. Besides, anti-IL-17 biologics are limited in that their effect is restricted to limiting IL-17 responses. In contrast, rIL-2 therapy has broader effects beyond dampening IL-17, such as bolstering the Treg–mediated immunosuppression and/or decreasing autoreactive Tfh cells, which are likely to synergize with Th17 suppression to further prevent immunopathology. In this regard, given that Tfh and Th17 cells are similarly regulated by the IL-2/STA5 and IL-6/STAT3 pathways, the aforementioned combinational therapy with STAT3 blocking agents and rIL-2 is likely to simultaneously target Tfh and Th17 cells efficiently. In summary, the inhibitory effects of IL-2 in Th17 cells, and its subsequent effects on Auto-Ab responses and systemic inflammation, need to be evaluated when considering IL-2-based therapies for the treatment of autoimmune disorders.

### IL-2 and DN T Cells

Despite the accumulating evidence supporting a pathogenic role for DN T cells, the exact mechanisms that regulate DN T cell homeostasis are unknown, and there are currently no therapies to selectively deplete DN T cells *in vivo*. Importantly, however, work from George Tsokos’s group demonstrates that treatment with an inducible recombinant adeno-associated virus vector encoding IL-2 significantly reduced the frequency of IL-17^+^ DN T cells in MRL/*lpr* mice, which was accompanied by reduced pathology and kidney infiltration (138). The effect of IL-2 on DN T cells is likely independent of the role of IL-2 in Treg cells, as treatment with IL-2 is complexed with the anti-IL-2 monoclonal JES6-1, which selectively target CD25-expressing Tregs did not affect DN T cells. Nevertheless, because DN T cells express neglectable levels of CD25 and CD122 and poorly phosphorylate STAT5 in response to IL-2, the authors suggest that the effect of IL-2 on DN T cells is indirect. In any case, whereas the exact mechanism by which IL-2 prevents DN T cell accumulation remains elusive, these studies demonstrate a critical role for IL-2 in preventing DN T cell expansion *in vivo*. Given the potential pathological role of these cells and their contribution to sustaining pathogenic Ab responses, the effects of low-dose rIL-2 immunotherapies on DN T cells should be carefully examined in future low-dose rIL-2 clinical trials.

## Concluding Remarks

In conclusion, we present here the rationale for using new therapeutic regimens based on the combination of low-dose rIL-2 with other biologics to achieve ideal immunosuppression and improved disease scores. Since the original observation that in the absence of IL-2 signaling mice develop catastrophic autoimmune disease, our knowledge of the complex intersection of multiple underlining conditions contributing to autoimmunity has grown to include multiple T cell populations in addition to Treg cells. Armed with the understanding that Tfh, Th17, and DN T cells play critical roles in autoimmune disease progression and that they are efficiently depleted after rIL-2 treatment, it is time to consider how to leverage the broad-ranging effects of rIL-2 therapy to synergistically induce Treg cell immunosuppression along with the Tfh/Th17/DN T cells axis to efficiently prevent inflammation and auto-Ab-mediated pathology in autoimmune patients without the undesired side effects associated to systemic immunosuppression.

While some studies suggest that B cells do not express CD25 and STAT5 signaling is dispensable for B cell maturation and function (177), IL-2 favors B cell survival and PC differentiation *in vitro* (178–180). These potential “*positive*” effects of IL-2 in B cells could, to some extent, compensate for the absence of T cell help and be detrimental in the context of B-cell mediated pathologies, particularly when administered for short periods of time. Therefore, though it is clear that low-dose rIL-2 therapies promote immunosuppression, the potential intrinsic effects of low-dose rIL-2 treatment in B cells should be carefully examined. Similarly, IL-2 inhibits the development of T follicular regulatory (TFR) cells (181–183), a particular subset of Tregs that express Bcl6 and CXCR5 and localize into the B cell follicles where they suppress Tfh and GC B cell responses (184–186). Mechanistically, IL-2 signaling induces Blimp-1 expression in conventional Tregs cells, thereby preventing them from up-regulating Bcl6 and becoming TFR cells. Given that TFR cells have a suppressive function in Tfh and GCs, the lack of these cells after low-dose rIL-2 could enhance pathogenic B cell responses. Corresponding with this idea, the absence of TFR cells favors the outgrowth of self-reactive B cell clones in some models (181, 185). Nevertheless, the role of TFR cells is more complex than initially expected, as, rather than inhibit, they promote GC and Ab responses in some models (187, 188). Besides, TFR cells express low levels of CD25 (181, 182). Thus, it is unlikely that rIL2 therapy will have a preferential impact on TFR cells. In any case, despite the putative adverse effects of IL-2, treatment with low-dose rIL-2 and IL-2/anti-IL-2 Ab complexes efficiently decreases anti-DNA Ab titers in NZB/W F1 mice (189) and hinders influenza-specific B cell responses in influenza-infected mice (114). These data support the view that, when used *in vivo, *the dominant effect of IL-2 in the B cell response is immunosuppression.

## Author Contributions

Both authors contributed equally. All authors contributed to the article and approved the submitted version.

## Funding

This work was supported by grants from the University of Alabama at Birmingham (UAB), the National Institutes of Health grant R01AI150664 to A. Ballesteros-Tato, and the Lupus Research Alliance Novel Research Award to A. Ballesteros-Tato.

## Conflict of Interest

The authors declare that the research was conducted in the absence of any commercial or financial relationships that could be construed as a potential conflict of interest.
